# Dynamic electrocardiogram changes are a novel risk marker for sudden cardiac death

**DOI:** 10.1093/eurheartj/ehad770

**Published:** 2023-11-13

**Authors:** Hoang Nhat Pham, Lauri Holmstrom, Harpriya Chugh, Audrey Uy-Evanado, Kotoka Nakamura, Zijun Zhang, Angelo Salvucci, Jonathan Jui, Kyndaron Reinier, Sumeet S Chugh

**Affiliations:** Center for Cardiac Arrest Prevention, Department of Cardiology, Smidt Heart Institute, Cedars-Sinai Medical Center, Advanced Health Sciences Pavilion, Suite A3100, 127 S. San Vicente Blvd., Los Angeles, CA 90048, USA; Center for Cardiac Arrest Prevention, Department of Cardiology, Smidt Heart Institute, Cedars-Sinai Medical Center, Advanced Health Sciences Pavilion, Suite A3100, 127 S. San Vicente Blvd., Los Angeles, CA 90048, USA; Center for Cardiac Arrest Prevention, Department of Cardiology, Smidt Heart Institute, Cedars-Sinai Medical Center, Advanced Health Sciences Pavilion, Suite A3100, 127 S. San Vicente Blvd., Los Angeles, CA 90048, USA; Center for Cardiac Arrest Prevention, Department of Cardiology, Smidt Heart Institute, Cedars-Sinai Medical Center, Advanced Health Sciences Pavilion, Suite A3100, 127 S. San Vicente Blvd., Los Angeles, CA 90048, USA; Center for Cardiac Arrest Prevention, Department of Cardiology, Smidt Heart Institute, Cedars-Sinai Medical Center, Advanced Health Sciences Pavilion, Suite A3100, 127 S. San Vicente Blvd., Los Angeles, CA 90048, USA; Division of Artificial Intelligence in Medicine, Department of Medicine, Cedars-Sinai Medical Center, Los Angeles, CA, Advanced Health Sciences Pavilion, Suite A3100, 127 S. San Vicente Blvd., Los Angeles, CA 90048, USA; Ventura County Health Care Agency, Ventura, CA, USA; Department of Emergency Medicine, Oregon Health & Science University, Portland, OR, USA; Center for Cardiac Arrest Prevention, Department of Cardiology, Smidt Heart Institute, Cedars-Sinai Medical Center, Advanced Health Sciences Pavilion, Suite A3100, 127 S. San Vicente Blvd., Los Angeles, CA 90048, USA; Center for Cardiac Arrest Prevention, Department of Cardiology, Smidt Heart Institute, Cedars-Sinai Medical Center, Advanced Health Sciences Pavilion, Suite A3100, 127 S. San Vicente Blvd., Los Angeles, CA 90048, USA; Division of Artificial Intelligence in Medicine, Department of Medicine, Cedars-Sinai Medical Center, Los Angeles, CA, Advanced Health Sciences Pavilion, Suite A3100, 127 S. San Vicente Blvd., Los Angeles, CA 90048, USA

**Keywords:** Sudden cardiac death, ECG, Risk prediction, Dynamic, General population

## Abstract

**Background and Aims:**

Electrocardiogram (ECG) abnormalities have been evaluated as static risk markers for sudden cardiac death (SCD), but the potential importance of dynamic ECG remodelling has not been investigated. In this study, the nature and prevalence of dynamic ECG remodelling were studied among individuals who eventually suffered SCD.

**Methods:**

The study population was drawn from two prospective community-based SCD studies in Oregon (2002, discovery cohort) and California, USA (2015, validation cohort). For this present sub-study, 231 discovery cases (2015–17) and 203 validation cases (2015–21) with ≥2 archived pre-SCD ECGs were ascertained and were matched to 234 discovery and 203 validation controls based on age, sex, and duration between the ECGs. Dynamic ECG remodelling was measured as progression of a previously validated cumulative six-variable ECG electrical risk score.

**Results:**

Oregon SCD cases displayed greater electrical risk score increase over time vs. controls [+1.06 (95% confidence interval +0.89 to +1.24) vs. −0.05 (−0.21 to +0.11); *P* < .001]. These findings were successfully replicated in California [+0.87 (+0.7 to +1.04) vs. −0.11 (−0.27 to 0.05); *P* < .001]. In multivariable models, abnormal dynamic ECG remodelling improved SCD prediction over baseline ECG, demographics, and clinical SCD risk factors in both Oregon [area under the receiver operating characteristic curve 0.770 (95% confidence interval 0.727–0.812) increased to area under the receiver operating characteristic curve 0.869 (95% confidence interval 0.837–0.902)] and California cohorts.

**Conclusions:**

Dynamic ECG remodelling improved SCD risk prediction beyond clinical factors combined with the static ECG, with successful validation in a geographically distinct population. These findings introduce a novel concept of SCD dynamic risk and warrant further detailed investigation.

## Structured Graphical Abstract

**Structured Graphical Abstract:**
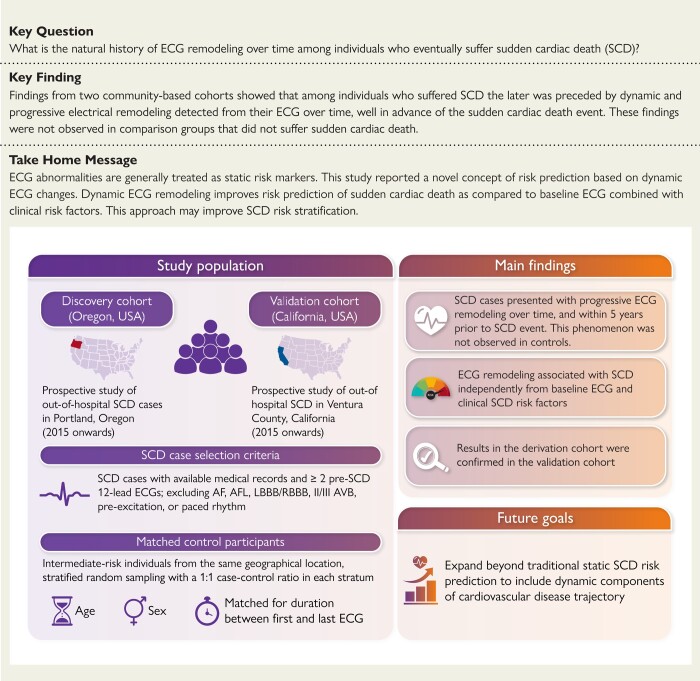
Summary of the study population, main findings, and future goals. AF, atrial fibrillation; AFL, atrial flutter; AVB, atrioventricular block; ECG, electrocardiogram; LBBB, left bundle branch abnormality; RBBB, right bundle branch abnormality; SCD, sudden cardiac death.

**See the editorial comment for this article ‘A paradigm change in sudden cardiac death risk prediction: ‘static' goes out, ‘dynamic' comes in’, by P.J. Schwartz and P. Cerea, https://doi.org/10.1093/eurheartj/ehae051.**

## Introduction

Heart disease remains a major cause of death worldwide, and ∼40% are sudden cardiac deaths (SCDs)^1,2^ highlighting the importance of prediction and prevention to reduce the burden of premature mortality due to SCD. Based on two landmark randomized clinical trials 20 years ago, patients with severely reduced left ventricular ejection fraction (LVEF <35%) receive primary prevention implantable cardioverter defibrillators.^3,4^ However, there is increasing recognition that LVEF <35% is an inadequate risk predictor,^5,6^ and there are currently no SCD risk stratification tools for individuals with LVEF >35%.

Substantial scientific efforts have been focused on identifying SCD risk factors from the routine 12-lead electrocardiogram (ECG) with the goal of extending SCD risk assessment beyond severely reduced LVEF. Prior studies have measured ECG abnormalities as static parameters, i.e. quantified ECG abnormalities at a given moment, and analysed the association with SCD risk.^7–9^ These studies have found several SCD risk markers on the static ECG, representing a wide range of abnormalities including resting heart rate, cardiac depolarization, and repolarization. We have previously developed and validated a cumulative six-variable ECG electrical risk score (ERS) that is independently associated with the risk of SCD.^7^ However, cardiovascular function changes over time, and current statistical approaches for ECG analysis are limited by the inability to capture dynamic changes in the cardiovascular disease risk trajectory. We hypothesized that progressive abnormalities in cardiac function over time also manifest as dynamic ECG remodelling, which could potentially augment the current approach to SCD risk stratification. We utilized data from two community-based SCD cohorts and matched controls to investigate and validate the possible occurrence of pre-SCD dynamic ECG remodelling measured as increase in the cumulative six-variable ECG ERS.

## Methods

### Discovery cohort

#### Sudden cardiac death cases

Sudden cardiac death cases in the discovery cohort were identified from the Oregon Sudden Unexpected Death Study (SUDS, since 2002), which is a prospective and ongoing community-based study of out-of-hospital cardiac arrest from the Portland, OR, USA, metro area (catchment population ∼1 million). Methods of the study have been described in detail previously.^5,10^ Briefly, all suspected out-of-hospital SCDs are identified in collaboration with multiple sources, including the region’s two-tiered emergency medical service (EMS) system, local hospital emergency departments, and the county medical examiner’s office. After identifying potential SCD cases from the area, three physician–researchers performed case adjudication for each potential SCD case based on a comprehensive evaluation of all available information including circumstances of death, medical records, medical examiner’s reports, and death certificates from Oregon state vital statistics records. Sudden cardiac death was defined as sudden, unexpected loss of the pulse due to a cardiac aetiology, and if the death was unwitnessed, participants were required to have been seen in their usual state of health in the previous 24 h. The study includes both successfully resuscitated cases and non-survivors. Patients with terminal illnesses or cases with likely non-cardiac causes of death (such as trauma/violent death, pulmonary embolism, aortic dissection, or overdose/substance use) were excluded.

For the present study, we included all SCD cases between February 2015 and January 2018 with at least two pre-SCD 12-lead ECGs and medical records available. All ECGs occurred prior to and unrelated to the SCD event. All survivors provided informed consent to review pre-SCD medical records, and for deceased participants, this requirement was waived.

#### Control participants

Control participants were recruited from the same geographical area (Portland, OR, metro area) to represent individuals at intermediate risk for SCD. Control participants were recruited from multiple sources, e.g. patients undergoing angiography or visiting outpatient cardiology clinics, or patients whose chest pain was assessed by EMS. Controls in our overall study were required to have no history of ventricular arrhythmias, but approximately half would have prevalent coronary artery disease (CAD), thus representing patients at ‘intermediate risk’ of SCD. The rationale for this is that the largest subgroup of SCD cases in the general population has associated significant CAD.^11^ Since our goal is to identify individuals at high risk of SCD (and not CAD), we frequency matched the two groups on prevalence of CAD. The control participants were selected before any knowledge of the results of their ECGs. Furthermore, for the purpose of this analysis, we frequency matched controls so that they would have a similar distribution of age, sex, and time between the two ECGs. Since controls did not have an index event analogous to the cases, ECGs that were performed during or after control recruitment could also be included. Stratified random sampling was used to select matched controls from the eligible patient population to achieve a 1:1 case–control ratio in each stratum. Matching was performed for age, sex, and duration between the first and last available ECGs. The size of each stratum was determined by the number of SCD cases in each sex, age (<18, 18–35, 35–55, 55–75, and >75 years old), and time between the ECG recording (1–5, 5–10, and >10 years) groups.

### Validation cohort

Sudden cardiac death cases in the validation cohort were identified from the Ventura Prediction of Sudden Death in Multi-ethnic Communities (PRESTO) study. The study protocol has been described previously.^12^ In brief, Ventura PRESTO (since 2015) is a prospective, ongoing, and population-based study ascertaining all out-of-hospital SCDs from Ventura County, CA, USA (catchment population ∼850 000), and uses identical case ascertainment, adjudication, and inclusion criteria to those used in the Oregon SUDS. For this validation cohort, we included all SCD cases between February 2015 and January 2022 with at least two pre-SCD 12-lead ECGs and medical records available. Both survivors and non-survivors of sudden cardiac arrest were included. All survivors provided informed consent, and this requirement was waived for deceased participants.

We selected validation control participants from outpatients of a regional healthcare system in southern California (Cedars-Sinai Medical Center) between 2012 and 2019. Similar to the discovery cohort, we prioritized a history of CAD diagnosis to achieve a comparable ‘intermediate-risk’ sample. Moreover, validation control participants were similarly matched to cases based on age, sex, and time between the two ECG recordings. Stratified random sampling was used to achieve a 1:1 case–control ratio in each stratum. Validation control participants were also selected before any knowledge of the results of their ECGs.

Data on comorbidities were collected from lifetime medical records for each case and control. History of myocardial infarction (MI) was defined as occurring before the most recent ECG for both cases and controls. For other comorbidities, data were collected prior to SCD (for cases) and up to the most recent visit (for controls).

**Figure 1:**
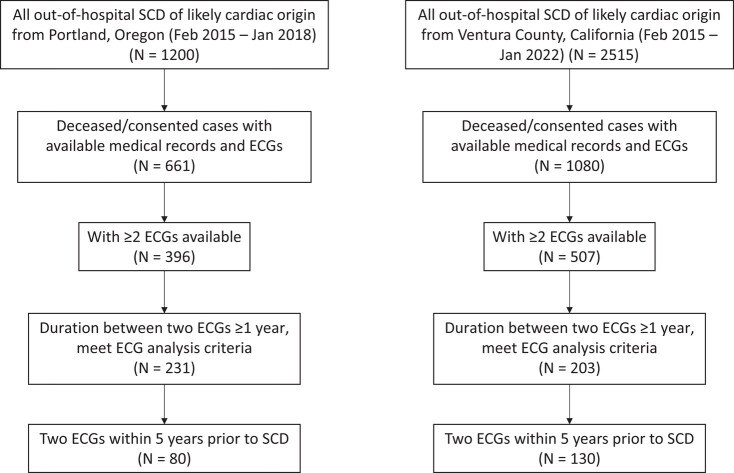
Sudden cardiac death (SCD) case selection. ECG, electrocardiogram.

The rationale for SCD case selection is presented in *Figure 1*.

Institutional review boards of Ventura County Medical Center, Oregon Health and Science University, Cedars-Sinai Health System, and all other relevant health systems and participating hospitals approved this study.

### Electrocardiogram analysis

All ECGs available in the lifetime health record were obtained for SCD cases (prior to ascertainment) and age-/sex-/time-matched control participants for the analysis with a paper speed of 25 mm/s and calibration of 10 mm/mV. An ECG ERS, previously developed and validated, was calculated for each ECG.^7^ If a subject had multiple pre-SCD ECGs available, we used the oldest (ECG1) and the most recent (ECG2). Electrocardiograms with atrial fibrillation or atrial flutter, left or right bundle branch abnormality (LBBB/RBBB), II/III degree atrioventricular block, pre-excitation, or paced rhythm were excluded from the analysis based on pre-specified ERS criteria. The ERS^7^ ranges from 0 to 6 based on the cumulative number of ECG abnormalities, with one point given for each parameter: resting heart rate >75 b.p.m., left ventricular hypertrophy (LVH) according to Sokolow–Lyon criteria, delayed QRS transition zone (≥V5), wide frontal QRS-T angle >90°, prolonged T_peak_-to-T_end_ (>89 ms) interval, and prolonged QTc (>450 ms for men and >460 ms for women).

### Statistical methods

Continuous variables are presented as mean (SD). Bivariate case–control comparisons of continuous and categorical variables were performed using independent sample *t*-tests and Pearson’s *χ*^2^ tests, respectively. All *P*-values are two sided. We performed a logistic regression to investigate whether ERS change over time is associated with SCD independently of baseline ERS, matching variables (age, sex, and the duration between ECGs), and clinical SCD risk factors [diabetes, hypertension, chronic renal insufficiency (CRI), COPD, sleep apnoea, seizure, syncope, obesity, and CAD]. Therefore, we included both baseline ERS and change in ERS in our models. In the models, ERS is a continuous variable. The odds ratio (OR) for the association between increased ERS and SCD reflects an increase of one unit in the ERS. The history of pre-SCD MI was excluded from the logistic regression because it was a subset of the history of CAD. We also performed an additional exploratory multivariable analysis using increases in abnormal QTc and T_peak_–T_end_ instead of the full ERS remodelling. We evaluated the performance of logistic regression models using the area under the receiver operating characteristic curve (AUC) across all possible sensitivity–specificity thresholds. Statistical analyses were performed with the IBM Statistical Package for Social Studies (SPSS) version 24.0.

### Sensitivity analysis

To investigate the ECG remodelling in the 5-year period before the SCD event, a subgroup analysis was performed with SCD cases who had both ECG1 and ECG2 within 5 years before the SCD event. These cases were selected from both discovery and validation SCD cohorts. By the above definition, the time between the most recent and the oldest ECG recordings did not exceed 5 years. Therefore, this subgroup of SCD cases was compared with a subgroup of control participants from both discovery and validation control cohorts who also had a duration between the most recent and the oldest available ECGs ≤ 5 years

### Mediation analysis

To evaluate potential causal pathways, and in particular whether changes in the ERS mediated sudden cardiac arrest (SCA) risk conferred by specific comorbidities, the relationships between comorbidities, ECG remodelling, and SCD event were tested by a causal mediation statistical framework implemented in the R package ‘mediation’.^13^ Briefly, the mediation analysis leveraged a potential outcome framework to partition the total effect from each exposure variable (in this case, each comorbidity) to the outcome variable (SCD event) as the sum of the causal mediation effect (mediated via ECG remodelling) and direct effect. Average causal mediation effects (ACME) were estimated by a model-based approach that sampled counterfactual outcomes; statistical inference was drawn against the null hypothesis where the ACME was zero.

We performed this statistical analysis with the pooled data from both cohorts using each comorbidity as the exposure variable if the comorbidity was significantly associated with SCD event (defined as *P* < .05 in *χ*^2^ test between cases and controls). This resulted in the inclusion of diabetes, hypertension, chronic obstructive pulmonary disease (COPD), CRI, and CAD in mediation analysis. We used a linear regression to model the exposure to mediator relationship and a logistic regression to model the exposure to outcome relationship. Age, sex, and ethnicity were not included as covariates in both models to control for potential confounding effects.

## Results

### Demographics and clinical characteristics

In both discovery and validation cohorts, SCD cases were matched to control participants based on age, sex, and duration between the two ECGs. In the discovery cohort (Oregon), we included a total of 231 SCD cases and 234 matched control participants. Sudden cardiac death cases had a mean age of 66.5 ± 13.6 years, 61% were male, and 27% were non-White individuals. Control participants had a mean age of 65.8 ± 11.1 years, 61% were male, and 14% were non-White individuals. Compared with controls, SCD cases were more likely to have diabetes (52% vs. 28%; *P* < .001), hypertension (87% vs. 74%; *P* < .001), CRI (49% vs. 17%; *P* < .001), COPD (29% vs. 9%; *P* < .001), and seizure (8% vs. 4%; *P* = .047).

For the validation cohort (California), we included 203 SCD cases (age: 70.3 ± 14.4 years, 54% male, and 41% non-White individuals) and 203 matched control participants (age: 68.4 ± 11.8 years, 54% male, and 56% non-White individuals). The prevalence of comorbidities was higher in SCD cases than in controls: diabetes (60% vs. 29%; *P* < .001), CRI (46% vs. 28%; *P* < .001), COPD (29% vs. 15%; *P* < .001), seizure (6% vs. 2%; *P* = .03), history of CAD (53% vs. 38%; *P* = .003), and history of MI prior to the most recent ECG (28% vs. 7%; *P* < .001). Controls were more likely to have sleep apnoea than SCD cases (23% vs. 11%; *P* = .002). All baseline characteristics in the discovery and the validation cohorts are described in *Table 1*.

**Table 1:** Study subject characteristics

	Oregon (discovery)			California (validation)		
	Case (*n* = 231)	Control (*n* = 234)	*P*-value	Case (*n* = 203)	Control (*n* = 203)	*P*-value
Male^a^, *n* (%)	141 (61%)	143 (61%)	.99	109 (54%)	109 (54%)	1.00
Female, *n* (%)	90 (39%)	91 (39%)		94 (46%)	94 (46%)	
Age, years, mean (SD)^a^	66.5 (13.6)	65.8 (11.1)	.60	70.3 (14.4)	68.4 (11.8)	.18
Ethnicity, *n* (%)			.006			<.001
White	168 (73%)	198 (86%)		120 (59%)	89 (44%)	
AA	33 (14%)	26 (11%)		3 (2%)	58 (28%)	
Hispanic	10 (4%)	2 (1%)		65 (32%)	33 (16%)	
Asian	11 (5%)	3 (1%)		13 (6%)	22 (11%)	
Others	9 (4%)	5 (1%)		2 (1%)	1 (1%)	
Duration between ECGs, years, median (IQR)^a^	5.1 (3.0–8.3)	5.4 (2.4–8.4)	.59	3.1 (1.9–4.6)	3.6 (2.4–4.7)	.83
Duration between the first ECG and SCD (cases) or enrolment (controls), years, median (IQR)	6.4 (4.2–10.0)	5.1 (1.5–9.4)	.001	4.2 (2.9–5.7)	5.1 (3.9–6.6)	.04
Diabetes, *n* (%)	121 (52%)	65 (28%)	<.001	121 (60%)	59 (29%)	<.001
Hypertension, *n* (%)	200 (87%)	172 (74%)	<.001	171 (84%)	159 (78%)	.13
CRI, *n* (%)	112 (49%)	39 (17%)	<.001	93 (46%)	57 (28%)	<.001
COPD, *n* (%)	67 (29%)	21 (9%)	<.001	58 (29%)	30 (15%)	<.001
Sleep apnoea, *n* (%)	60 (26%)	55 (24%)	.54	23 (11%)	47 (23%)	.002
Seizure, *n* (%)	19 (8%)	9 (4%)	.047	13 (6%)	4 (2%)	.03
Syncope, *n* (%)	21 (9%)	11 (5%)	.06	25 (12%)	21 (10%)	.53
Obesity, *n* (%)	109 (47%)	106 (45%)	.68	80 (39%)	71 (35%)	.36
History of CAD, *n* (%)	131 (57%)	113 (48%)	.07	107 (53%)	77 (38%)	.003
History of MI, *n* (%)	60 (26%)	74 (32%)	.18	56 (28%)	14 (7%)	<.001

AA, African American; CRI, chronic renal insufficiency; COPD, chronic obstructive pulmonary disease; CAD, coronary artery disease; IQR, interquartile range; MI, myocardial infarction.

^a^SCD cases and control participants were frequency matched by sex, age, and duration between the two ECGs.

### Multivariate models for sudden cardiac death risk

To investigate risk factors that are independently associated with the SCD event, we performed a multivariable logistic regression including all baseline comorbidities (*Table 1*), baseline ERS, and increased ERS. In multivariable analysis models for both discovery and validation cohorts, a one-unit increase in the ERS was independently associated with SCD event {Oregon: OR 3.2 [95% confidence interval (CI) 2.49–4.1]; *P* < .0001; California: OR 3.24 [95% CI 2.48–4.24]; *P* < .0001}. Detailed results of the multivariable model are described in *Table 2* for both discovery and validation cohorts.

**Table 2:** Univariate and multivariate models for sudden cardiac death risk factors

	Oregon				California			
	Unadjusted OR (95% CI)	*P*-value	Adjusted OR (95% CI)	*P*-value	Unadjusted OR (95% CI)	*P*-value	OR (95% CI)	*P*-value
Age	1 (0.99–1.02)	.6	1.01 (0.99–1.04)	.17	1.01 (1–1.03)	.16	1 (0.98–1.02)	.93
Female sex	1 (0.69–1.46)	.99	0.64 (0.39–1.07)	.09	1 (0.68–1.48)	1	1.51 (0.87–2.61)	.14
Duration between ECGs	0.99 (0.95–1.03)	.59	0.94 (0.89–1)	.05	1.01 (0.92–1.11)	.82	1.03 (0.91–1.16)	.67
Diabetes	2.86 (1.95–4.2)	<.0001	1.73 (1–3)	.05	3.6 (2.38–5.44)	<.0001	3.57 (2.01–6.33)	<.0001
Hypertension	2.33 (1.44–3.75)	.0005	0.96 (0.5–1.85)	.91	1.48 (0.89–2.45)	.13	0.79 (0.38–1.66)	.53
CRI	4.71 (3.06–7.23)	<.0001	3.09 (1.77–5.39)	<.0001	2.17 (1.43–3.27)	.0002	1.35 (0.75–2.43)	.32
COPD	4.14 (2.44–7.05)	<.0001	3.44 (1.78–6.65)	.0002	2.31 (1.41–3.78)	.0009	2.63 (1.36–5.11)	.0042
Sleep apnoea	1.14 (0.75–1.74)	.54	0.81 (0.45–1.46)	.48	0.42 (0.25–0.73)	.002	0.23 (0.11–0.5)	.0002
Seizure	2.24 (0.99–5.06)	.05	1.27 (0.45–3.61)	.66	3.4 (1.09–10.62)	.03	4 (0.97–16.51)	.05
Syncope	2.03 (0.95–4.31)	.07	2.42 (0.92–6.42)	.07	1.22 (0.66–2.25)	.53	1.23 (0.53–2.88)	.63
Obesity	1.08 (0.75–1.55)	.68	0.69 (0.41–1.18)	.17	1.21 (0.81–1.81)	.36	1.42 (0.79–2.57)	.24
History of CAD	1.4 (0.97–2.02)	.07	0.89 (0.54–1.46)	.63	1.82 (1.23–2.71)	.0029	1.11 (0.63–1.96)	.71
Baseline ERS	1.37 (1.16–1.63)	.0002	2.88 (2.16–3.83)	<.0001	1.61 (1.33–1.95)	<.0001	3.26 (2.42–4.4)	<.0001
Increased ERS	1.97 (1.66–2.33)	<.0001	3.2 (2.49–4.1)	<.0001	1.99 (1.65–2.4)	<.0001	3.24 (2.48–4.24)	<.0001

Age (years), duration between ECGs (years), baseline ERS (six-variable ECG electrical risk score), and increased ERS (change in the six-variable ERS) are provided as continuous variables, and the ORs represent the increase in odds for a one-unit increase. The OR for the association between baseline ERS and increased ERS and SCD reflects an increase of one unit in the ERS. The other variables are provided as categorical variables.

CRI, chronic renal insufficiency; COPD, chronic obstructive pulmonary disease; CAD, coronary artery disease; MI, myocardial infarction; ERS, electrical risk score.

**Figure 2:**
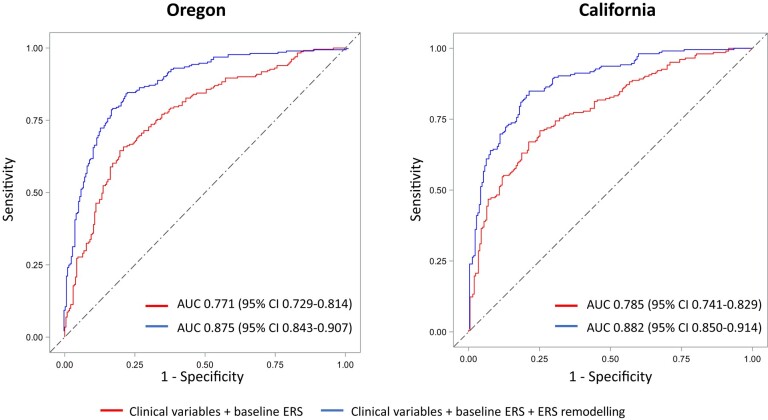
Utility of dynamic electrocardiogram remodelling beyond baseline electrocardiogram and clinical risk factors. Receiver operating curves for the identification of sudden cardiac death cases in the discovery (Oregon) and validation (California) cohorts with logistic regression models. Clinical variables include diabetes, hypertension, chronic renal insufficiency, chronic obstructive pulmonary disease, sleep apnoea, seizure, syncope, obesity, and coronary artery disease. AUC, area under the receiver operating curve; ERS, electrical risk score.

To evaluate the predictive power of abnormal ERS progression beyond baseline ERS and clinical SCD risk factors, we performed logistic regression analyses including baseline ERS and clinical variables with and without ERS change in both Oregon and California cohorts. In Oregon, addition of the ERS change to baseline ERS and clinical variables improved the discriminative value of SCD from an AUC of 0.771 (0.729–0.814) to an AUC of 0.875 (0.843–0.907). Similar results were obtained in the California cohort, in which addition of the ERS change improved the AUC from 0.785 (0.741–0.829) to 0.882 (0.850–0.914). Regression model performance metrics and AUC curves in Oregon and California cohorts are presented *Figure 2*.

### Electrocardiogram remodelling in sudden cardiac death cases vs. control participants

**Figure 3:**
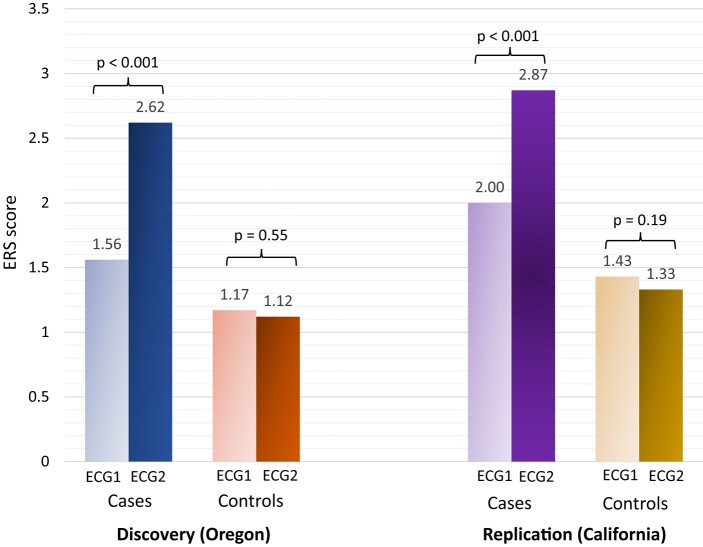
Electrocardiogram remodelling in sudden cardiac death cases vs. control participants. In Oregon, 231 sudden cardiac death cases were matched to 234 controls by age, sex, and duration between two electrocardiograms. In California, 203 sudden cardiac death cases were matched to 203 controls by age, sex, and duration between two electrocardiograms. The difference in the electrical risk score progression was statistically significant between sudden cardiac death cases and controls in both discovery and validation cohorts. Electrical risk score 1 and electrical risk score 2 refer to electrocardiogram-based electrical risk scores for the oldest and the most recent electrocardiogram, respectively. ECG, electrocardiogram.

**Figure 4:**
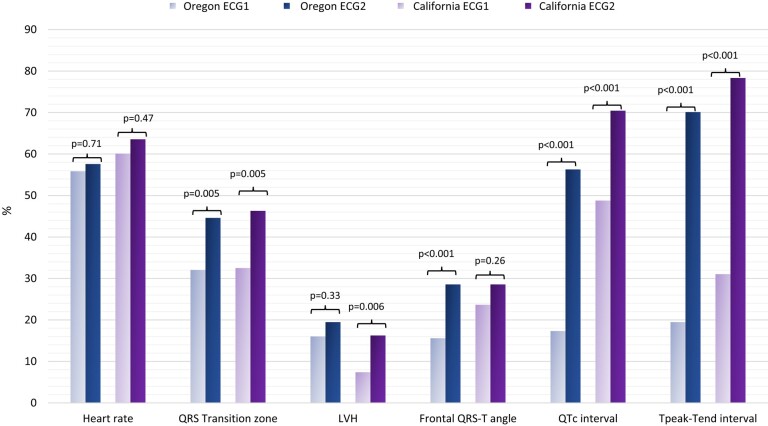
Changes in the prevalence of each electrical risk score component in sudden cardiac death cases. LVH, left ventricular hypertrophy; ECG1, the oldest ECG; ECG2, the most recent ECG.

In both cohorts, the duration between the most recent and oldest ECGs was matched between cases and control participants, with a mean 6.0 ± 4.0 years vs. 6.2 ± 4.5 years, respectively (Oregon), and a mean 3.7 ± 2.6 years vs. 3.7 ± 1.6 years, respectively (California). Electrical risk score progression was significantly higher among SCD cases in comparison to controls in both discovery and validation cohorts: Oregon +1.06 (95% CI +0.89; +1.24) vs. −0.05 (95% CI −0.21; +0.11; *P* < .001) and California +0.87 (95% CI +0.7; +1.04) vs. −0.11 (95% CI −0.27; 0.05; *P* < .001), respectively (*Figure 3*). Sudden cardiac death cases also had higher ERS scores than controls at baseline (ECG1) in both cohorts: 1.56 (95% CI 1.41–1.71) vs. 1.17 (95% CI 1.04–1.30); *P* < .001 in Oregon and 2.00 (95% CI 1.84–2.16) vs. 1.43 (95% CI 1.29–1.57); *P* < .001 in California. The main ECG parameters driving the increase of ERS for SCD cases in both cohorts were prolonged QTc (Oregon: from 17% to 56%; *P* < .001; California: from 48% to 70%; *P* < .001) and prolonged T_peak_–T_end_ interval (Oregon: from 19% to 70%; *P* < .001; California: from 31% to 78%; *P* < .001). At baseline, increased heart rate was the most common ECG abnormality in both cohorts but did not show statistically significant changes over time (*Figure 4*).

Because we observed that increases in the proportion of individuals with abnormal QTc and T_peak_ were the main drivers of change in the ERS over time, we performed an exploratory analysis using increases in abnormal QTc and T_peak_–T_end_ (as opposed to the full ERS) in our multivariable model and found that the AUC for that model performed comparably to the model with the full ERS in the internal [AUC = 0.881 (0.850–0.912)] and external cohorts [AUC = 0.861 (0.825–0.897)].

### Subgroup analysis of electrocardiograms 5 years before the sudden cardiac death event

To investigate the temporal association between dynamic ECG remodelling and SCD, we performed a subgroup analysis with SCD cases who had both ECG1 and ECG2 within 5 years before the SCD event. We compared this subgroup of SCD cases with matched controls. In Oregon, 80 SCD cases were matched with 104 controls by age (63.8 ± 14.3 vs. 64.3 ± 12.8 years), sex (male 65% vs. 63%), and duration between two ECGs (2.6 ± 1.1 vs. 2.5 ± 1.2 years). In California, 130 SCD cases were matched with 162 controls by age (69.2 ± 14.6 vs. 68.0 ± 11.9 years) and sex (male 55% vs. 56%), but the duration between two ECGs was slightly longer in California controls than in cases (3.1 ± 1.1 vs. 2.5 ± 1.0 years; *P* < .001).

**Figure 5:**
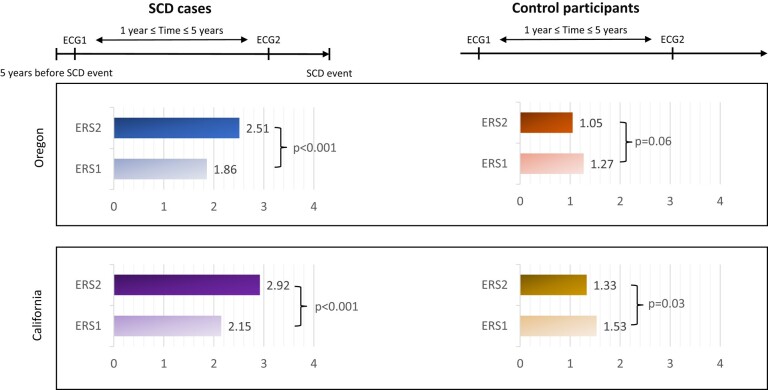
The progression of electrical risk score within 5 years before sudden cardiac death in comparison to matched controls. In Oregon, 80 sudden cardiac death cases were matched with 104 controls by age, sex, and duration between two electrocardiograms. In California, 130 sudden cardiac death cases were matched with 162 controls by age and sex. The duration between two electrocardiograms was slightly longer in California controls than in cases (3.1 ± 1.1 vs. 2.5 ± 1.0 years; *P* < .001). The difference in the electrical risk score progression was statistically significant between sudden cardiac death cases and controls in both discovery and validation cohorts. The values represent the mean changes in electrical risk score over time (e.g. +0.65 means an increase of 0.65 units in the electrical risk score from the oldest electrocardiogram to the most recent electrocardiogram). ECG1, the oldest ECG; ECG2, the most recent ECG; ERS1 and ERS2, ECG-based electrical risk score for ECG1 for ECG2, respectively.

Within 5 years before the SCD events, SCD cases from both discovery and validation cohorts had higher baseline ERS in comparison to controls: 1.86 (95% CI 1.57–2.15) vs. 1.27 (95% CI 1.06–1.48); *P* < .001 in Oregon and 2.15 (95% CI 1.95–2.34) vs. 1.53 (95% CI 1.38–1.68); *P* < .001 in California. In both discovery and validation cohorts, SCD cases also displayed a higher increase in ERS than controls: +0.65 (95% CI +0.34 to +0.96) vs. −0.22 (95% CI −0.45 to +0.01); *P* < .001 in Oregon and +0.77 (95% CI 0.56 to 0.97) vs. −0.2 (95% CI −0.37 to −0.02); *P* < .001 in California (*Figure 5*).

### Mediation analysis

**Figure 6:**
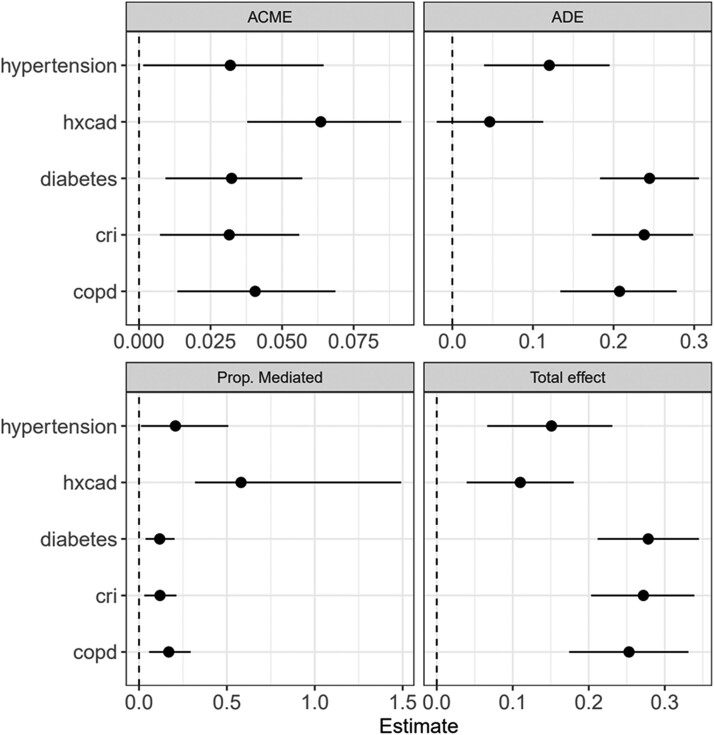
Mediation analysis of comorbidity effect on sudden cardiac death mediated via electrical risk score. Results are presented as effect sizes for the association of each comorbidity with sudden cardiac death. Effect sizes are estimates of the increase in the probability of sudden cardiac death comparing individuals with and without each comorbidity. hxcad, coronary artery disease; cri, chronic renal insufficiency; copd, chronic obstructive pulmonary disease; ACME, average causal mediation effect (effect of comorbidity on SCD mediated via ERS); ADE, average direct effect (direct effect of comorbidity, not explained by ERS); Total effect, =ACME + ADE (the overall effect of comorbidity on SCD).

In the mediation analysis using the pooled data from two cohorts, only a moderate proportion of the effects of five important comorbidities (hypertension, diabetes, CRI, COPD, and CAD) on SCD event were mediated through increased ERS: 20.7% (95% CI 11.7%–50.8%) for hypertension (*P* = .045), 11.8% (95% CI 3.8%–20.2%) for diabetes (*P* = .004), 11.9% (95% CI 3.0%–21.3%) for CRI (*P* = .01), and 16.9% (95% CI 5.8%–29.4%) for COPD (*P* = .002). History of CAD had the highest mediated proportion of the indirect effect on SCD via increased ERS: 58.1% (95% CI 31.9%–149.1%); *P* < .001. The findings of the mediation analysis are presented in *Figure 6*.

## Discussion

In this study, we aimed to investigate pre-SCD ECG remodelling in two population-based SCD cohorts (Oregon and California). In both cohorts, SCD cases had a higher baseline ERS compared to controls and a significant increase in ERS prior to the SCD event. This ECG remodelling was also temporally associated with SCD during the 5 years preceding the event in both cohorts. Abnormal ECG remodelling improved SCD prediction over baseline ECG and clinical risk factors in both cohorts. The main drivers of the ERS increase were prolonged QTc and T_peak_–T_end_ interval, and our sensitivity analysis indicated that a combination of these two variables alone may be adequate to identify ECG changes over time that are clinically important for dynamic SCA risk. Only a moderate proportion of the effects of clinical SCD risk factors were mediated through increased ERS, and the proportion was highest for previously diagnosed CAD. These findings introduce a novel concept of ‘dynamic SCD risk’ and suggest that dynamic ECG remodelling may be temporally associated with an increased risk of SCD, with the potential to augment the current “static” approach to SCD risk stratification (*Structured Graphical Abstract*).

To the best of our knowledge, this is the first study to investigate and validate dynamic ECG changes in association with out-of-hospital SCD. A seminal study by Schwartz and Wolf from 1978 found that the average QTc and high QT variability from multiple ECGs over time associates with the risk of sudden death in patients with MI, thereby incorporating temporal changes for the first time.^14^ Several other SCD risk markers from the static 12-lead ECG have been identified in subsequent studies.^7–9,15^ Single ECG risk markers usually have low predictive power, and we have previously published a six-variable ECG risk score that utilized several ECG abnormalities to improve SCD prediction accuracy.^7^ However, prior studies have utilized static single measurements and therefore not able to evaluate the impact of the progression of individual ECG markers or a combination of these. Sudden cardiac death is a relatively rare event at the community level (∼50/100 000 person-years), and it is challenging to obtain archived pre-SCD ECGs from a feasible number of cases. Prospective ascertainment of out-of-hospital SCDs from two community-based cohorts with a total catchment population of 1.85 million from two geographically distinct sites made this study feasible. As opposed to most community-based out-of-hospital cardiac arrest registries, we were able to collect lifetime pre-SCD medical records and capture all available archived 12-lead ECG recordings that were performed in advance of the SCD event. Despite the demographic and clinical differences between SCD cases from Oregon and California, the study findings were replicated in the external cohort with almost identical results. Therefore, these results are likely to reflect a real association and are unlikely to be confounded by any specific aspects of either cohort (e.g. patient demographics, clinical profiles, and control participants).

Selection of control participants presents a challenge in population-based studies of SCD. Our approach of enrolling individuals at intermediate risk of SCD, many with pre-existing CAD, allowed us to efficiently control for CAD in multivariable analyses. Overall, SCD cases were less healthy than control participants, since many cardiovascular and non-cardiac risk factors are associated with the risk of SCD. Although we adjusted for these risk factors in multivariable models, there may be other differences we did not measure and for which we could not adjust. Ideally, our conclusions should be replicated in a prospective cohort study.

In the present study, the most important drivers of the ERS remodelling were prolonged QTc and T_peak_–T_end_ interval. We have previously reported (from the Oregon population) that both diabetes mellitus and QT-prolonging drugs are significant predictors of prolonged QTc and increased risk of SCD.^16^ However, QT prolongation in the absence of diabetes or QT-prolonging drugs was an even stronger predictor of SCD, resulting in a five-fold increase in SCD risk among patients with CAD. The present study represents a real-world scenario and demonstrates that QTc prolongation is an important driver of dynamic risk of SCD, and further investigations are needed to clarify which determinants for QTc prolongation over time associate with the highest dynamic risk of SCD.

These results add to emerging evidence regarding the potential utility of dynamic markers for cardiovascular event risk stratification. A recent study reported that evaluating trends of QRS duration, QTc, RR, and ST on continuous telemetry monitoring within a 3-h window can identify patients at risk of in-hospital cardiac arrest, independent of other patient data.^17^ Moreover, analysing heart rate variability or intracardiac J-point elevation on implantable cardioverter defibrillators could improve the near-term prediction of malignant ventricular arrhythmias in patients with ischaemic cardiomyopathy.^18–20^ However, the clinical applicability of these findings is limited due to the need for continuous monitoring. The standard resting 12-lead ECG can offer a widely available and inexpensive method to augment risk stratification in large and diverse patient groups using dynamic ECG remodelling over time. Our mediation analysis demonstrated that the effect of CAD on the risk of SCD was largely mediated through electrical remodelling manifested on the ECG, suggesting that dynamic changes on the standard resting 12-lead ECG may be a reasonable tool for a preliminary risk assessment of CAD-related SCD.

Our findings suggest that SCD cases present with a form of escalated abnormal cardiac electrical trajectories that manifest as ECG remodelling. This potentially reflects a deterioration of the underlying cardiac disease (SCD substrate) which may associate with a temporally increased risk of SCD. The concept of ‘dynamic SCD risk’ in the general population can be linked to the hypothesis that adverse progression of the underlying cardiac substrate is an independent marker of SCD risk, manifesting as an increase in ERS. For example, previous studies have demonstrated that the risk of SCD is increased during myocardial scar formation and remodelling which occurs within the first 30 days following an MI.^6,21–23^

Current risk prediction of SCD is based on the evaluation of long-term risk using static biomarkers, especially LVEF (<35%). However, this approach has been proven to be inadequate and also does not identify at least 70% of SCD cases who have LVEF >35% prior to the event.^5^ Therefore, there is a significant need to improve and extend SCD primary prevention beyond the use of this single long-term static risk marker. In the future, a more comprehensive assessment of SCD risk could potentially be expanded beyond baseline clinical profiling, also including the evaluation of disease progression. Identification of high-risk cardiac disease trajectories has the potential for improving sensitivity of SCD risk assessment while remaining specific. The results of our study suggest that addition of dynamic SCD risk markers that reflect risk progression over time to traditional static risk markers could augment SCD risk prediction.

### Limitations

This study has some limitations that should be considered while interpreting the findings. The analysis was restricted to SCD cases with at least two available ECGs prior to and unrelated to the SCD event, which could lead to selection bias. In addition, the retrospective nature of the pre-SCD data collection could have led to missing data on pre-arrest clinical characteristics. Given that we did not have a separate cohort of non-SCD, we cannot conclude whether the ERS remodelling is a specific risk factor for SCD. Lastly, we were not able to access the indications for repeat ECG recordings. However, our results were validated in a geographically distinct SCD cohort from California, with almost identical results, which mitigates the possibility of a chance finding.

## Conclusions

Sudden cardiac death cases presented with abnormal ECG remodelling prior to the SCD event, whereas matched controls had stable ECG risk scores over a similar time period. Abnormal ECG remodelling added predictive value over baseline ECG and clinical SCD risk factors, and the results were validated in a geographically distinct SCD cohort. This novel concept and these findings warrant further detailed investigation to augment current static SCD risk stratification strategies, by incorporating dynamic components of the cardiovascular disease progression trajectory.

## Data Availability

Data will be made available upon reasonable request.

## Pre-registered Clinical Trial Number

None supplied.
